# Prevalence of Vertebral Fractures and Their Prognostic Significance in the Survival in Patients with Chronic Kidney Disease Stages 3‒5 Not on Dialysis

**DOI:** 10.3390/jcm9051604

**Published:** 2020-05-25

**Authors:** Cristina Castro-Alonso, Luis D’Marco, Jaume Pomes, Monserrat Del Amo Conill, Ana Isabel García-Diez, Pablo Molina, María Jesús Puchades, José Manuel Valdivielso, Verónica Escudero, Jordi Bover, Juan Navarro-González, Begoña Ribas, Luis Manuel Pallardo, José Luis Gorriz

**Affiliations:** 1 Department of Nephrology, Hospital Universitario Dr. Peset, FISABIO, Universidad de Valencia, 46017 Valencia, Spain; cristina.c.med@gmail.com (C.C.-A.); molina_pab@gva.es (P.M.); veesque@gmail.com (V.E.); pallardo_lmi@gva.es (L.M.P.); 2 Department of Nephrology, Hospital Clínico Universitario, INCLIVA, Universidad de Valencia, 46010 Valencia, Spain; luisgerardodg@hotmail.com (L.D.M.); chuspuchades@gmail.com (M.J.P.); 3 Department of Radiology, Hospital Clínic, 08036 Barcelona, Spain; JPOMES@clinic.cat (J.P.); MDELAMO@clinic.cat (M.D.A.C.); AIGARCIA@clinic.cat (A.I.G.-D.); 4 Vascular and Renal Translational Research Group, IRBLleida, RedinRen-ISCIII, 25198 Lleida, Spain; valdivielso@medicina.udl.cat; 5 Department of Nephrology, Fundació Puigvert, IIB Sant Pau, RedinRen, 08025 Barcelona, Spain; jbover@fundacio-puigvert.es; 6 Research Unit and Nephrology Service, University Hospital Nuestra Señora de Candelaria, Santa Cruz de Tenerife and Instituto de Tecnologías Biomédicas, Universidad de La Laguna, 38010 Tenerife, GEENDIAB REDINREN, Spain; jnavgon@gobiernodecanarias.org; 7 Department of Nephrology, Hospital La Paz, 28046 Madrid, Spain; bego_rivas@hotmail.com

**Keywords:** chronic kidney disease, fractures, vascular calcification

## Abstract

*Background*: The prevalence of vertebral fractures (VF) and their association with clinical risk factors and outcomes are poorly documented in chronic kidney disease (CKD) cohorts. The aim of the study was to evaluate the prevalence of VF in patients with non-dialysis dependent CKD (NDD-CKD), their value in predicting mortality and its correlation with parameters of bone mineral metabolism and vascular calcification. *Materials and Methods*: 612 NDD 3‒5 stage CKD patients participating in the OSERCE-2 study, a prospective, multicenter, cohort study, were prospectively evaluated and categorized into two groups according to presence or absence of VF at enrollment. VF were assessed with lateral radiographs and Genant semi-quantitative method was applied. Three radiologists specialized in musculoskeletal radiology performed consensual reading of individual images obtained using a Raim DICOM Viewer and a Canon EOS 350 camera to measure with Java Image software in those who had traditional acetate X-ray. Factors related to VF were assessed by logistic regression analysis. Association between VF and death over a 3-year follow-up was assessed by Kaplan-Meier survival curves and Cox-proportional hazard models. *Results*: VF were detected in 110 patients (18%). Serum phosphate levels (OR 0.719, 95% CI 0.532 to 0.972, *p* = 0.032), ankle-brachial index < 0.9 (OR 1.694, 95% CI 1.056‒2.717, *p* = 0.029) and treatment with bisphosphonates (OR 5.636, 95% CI 1.876‒16.930, *p* = 0.002) were independently related to the presence of VF. After a median follow-up of 35 months (IQR: 17‒37 months), 62 patients (10%) died. The causes of death were cardiovascular (*n* = 21, 34%) and infectious (*n* = 11, 18%). In the crude analysis, fractured patients group had poorer survival (log-rank test, *p* = 0.02). After multivariate adjustment for age, MDRD, albumin, diabetes mellitus, comorbidity, Adragao Score > 3 and serum phosphate, the presence of VF (HR 1.983, 95% CI 1.009‒3.898, *p* = 0.047) were an independent predictor of all-cause mortality. *Conclusions*: In our study 18% of patients with NDD-CKD have VF. Factors associated with VF were age, low serum phosphate levels and peripheral vascular disease. The presence of VF was an independent risk factor for mortality in stages 3‒5 NDD-CKD patients. Clinical trials are needed to confirm whether this relationship is causal and reversible with treatment for osteoporosis.

## 1. Introduction

Fractures are an important and debilitating condition, mainly in older people and particularly in women. Although osteoporosis is the main risk factor for fractures in the general population, renal osteodystrophy and “uremic” osteoporosis associated with a reduced glomerular filtration rate (GFR) have also been implicated [1]. Importantly, in patients with chronic kidney disease (CKD), mineral and bone disorder (CKD-MBD) related to abnormalities of calcium and phosphate metabolism and bone formation/turnover dysregulation (renal osteodystrophy) are also associated with an increased risk. Thus, CKD patients are at increased risk of hip and vertebral fractures (VF) [2,3]. However, hip fracture is the most common type among end stage renal disease (ESRD) patients; although other studies have found a high incidence of this type of fracture among patients with progressive CKD [4,5,6].

The epidemiological data varies among stages of CKD with diverse and even controversial results regarding incidence, prevalence and risk factors, at least partially due to different screening policies [7,8]. Nevertheless, patients on dialysis have worse prognosis and they also experience fractures at a younger age with higher mortality rates following a fracture compared to those patients without CKD [9]. To date, a few studies have examined the risk of fracture in patients with non-dialysis dependent (NDD) CKD [5,10,11]. Whereas hip fractures are rarely undiagnosed, many VFs are asymptomatic or cause mild pain, being not diagnosed until the fracture is associated with severe pain [12]. The prevalence of asymptomatic VFs among CKD patients is estimated between 2‒28% [7].

The association of VFs with clinical risk factors and worse outcomes are also poorly documented in CKD cohorts. Age, sex and the presence of diabetes have been described as common risk factors for new fractures [3,13,14]. Besides this, the role of bone and mineral disorders, the metabolism of 25-hydroxyvitamin D and PTH levels have been of recent interest and still scarcely explored in this population [14,15,16,17]. In the recent literature the impact of vascular disease on bone pathology has been emphasized. Recent data from general population and CKD patients has shown an inverse relationship between cardiovascular morbidity and mortality, vascular calcification and bone mineral density the pathogenic mechanisms and factors involved remain to be clarified. Additionally, it is increasingly acknowledged the narrow relationship between bone remodeling and vascular calcification (the so-called bone-vascular cross-talk) both in CKD patients and the general population [18,19]. Moreover, few studies have demonstrated an association between bone fractures and an increased morbidity and mortality among dialysis patients [20,21,22]. However, these possible associations remain to be further explored in NDD-CKD patients. 

In this context, this study aims to explore the prevalence of VF in patients with stages 3‒5 NDD-CKD, the possible impact on survival, and investigate the relationship between parameters of renal disease, bone-mineral disorder and vascular calcification with the presence of VF. For this purpose, a *post-hoc* analysis of the OSERCE 2 study data was performed [23]. 

## 2. Material and Methods

This study of mineral and bone disorders in CKD describes the results of a *post-hoc* analysis of the observational prospective Estudio sobre las alteraciones del metabolismo OSeo-mineral en la Enfermedad Renal Cronica en España (OSERCE) 2 study carried out from May 2009 to May 2012 [23]. Thirty-nine hospitals participated in this 3-year follow-up multicenter investigation aimed to assess the prevalence of vascular calcification, its correlation with parameters of bone mineral metabolism, and its effect on mortality, hospitalization, and progression of renal failure after a follow-up period of 3 years in patients with NDD-CKD. OSERCE 2 study received the approval of the Institutional Review Board of Hospital Universitario Dr. Peset (Valencia) and all patients signed the informed consent forms for their inclusion, consistent with the principles of the Declaration of Helsinki. The study included 742 CKD patients with aged ≥18 years in NDD-CKD stages 3–5 (estimated GFR (eGFR) < 60 mL/min per 1.73 m^2^). The exclusion criteria were acute renal failure, life expectancy <12 months, and hospital admission during the month before inclusion. Researchers recruited consecutively the first 20 patients in each center. 

**Figure 1:**
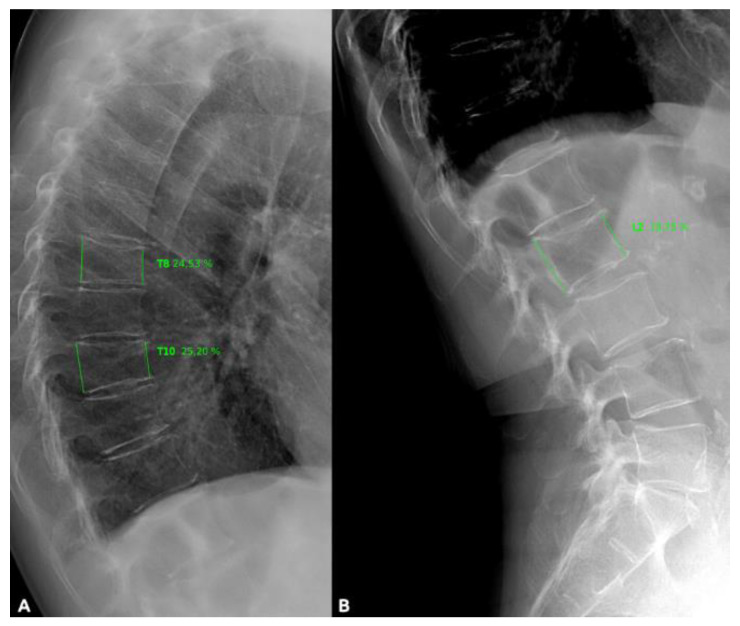
(**A**). Lateral thoracic radiograph showing (green lines) vertebral fractures in T-8 and T-10 with >20% of change in shape, size and height of vertebral body. (**B**). Normal vertebrae (L2) without pathological changes (green lines). T, thoracic vertebrae; L, lumbar vertebra.

Lateral full spine X-ray was available in 612 patients representing the final sample of this sub analysis. A semiquantitative approximation of Genant was used to determine VF (Figure 1) [24]. Assessment of the images were independently performed by three experts in musculoskeletal radiology without access to clinical data of the patients. Consensus was sought in case of inconsistency in the readings or doubt, generally caused by transition anomalies. Thus, in the 355 (58%) patients with digital images, measurement was quantified using a Raim DICOM Viewer imaging system and in the others 257 (42%) with traditional acetate X-ray was convert to digital image using a Canon EOS 350 camera and measure with Java Image software (http://imagej.nih.gov/ij/). A VF was described as a change in the shape and size of the vertebral body, with a reduction in height comparing with expected vertebral height or that of the adjacent vertebra, considering fracture criteria a height loss of ≥20%. The Genant method allows one to determine the severity of VF, where mild deformity is classified as a 20–25% height loss (anterior, middle, and/or posterior relative to adjacent vertebrae); moderate a 25–40% height loss, and severe > 40% height loss) and differentiating VF from other, nonfracture deformities (Figure 1) [24]. 

Baseline visit included routine clinical anamnesis, details on current medication, ankle-brachial index (ABI) measurement, centralized laboratory analysis (serum calcium, phosphate, intact PTH (chemoluminiscence; Immulite 2000), calcidiol and calcitriol levels (radioimmunoassay), high sensitivity C-reactive protein (hsCRP), and creatinine, among other routine parameters). Abdomen, pelvis and hands X rays were performed to calculate the Adragao and Kauppila scores for vascular calcification [25,26]. The progression of kidney function was monitored with creatinine levels and eGFR determined at baseline and at months 12, 24, and 36, respectively. The eGFR was calculated using the Modification of Diet in Renal Disease (MDRD) formula [27]. Death event and its cause were recorded as main outcome.

## 3. Statistical Analysis

This sub-analysis included all patients with available full spine X-ray in the OSERCE 2 study. Therefore, sample size depended on the previous recruitment of OSERCE 2 and was determined by the availability of full spine X-ray to obtain the primary variable [23]. The results of the continuous variables are expressed as the mean ± SD or median (interquartile range [IQR]) as appropriate. Given that Kauppila and Adragao scores did not present a normal distribution, both vascular calcification scores were grouped together in dichotomous variables according to the presence or absence of prominent vascular calcifications (AS > 3 or KS > 6 as previously reported) [23]. The univariate analysis was undertaken using the Student *t*-test, Mann–Whitney *U*, or chi-squared test as appropriate. Factors independently related to presence of VF were assessed by logistic regression analysis, including those variables that were significant in a univariate analysis. Overall survival was estimated using the Kaplan-Meier method, and the log-rank test was used to compare the survival curve. The univariate and multivariate association between VF and death was assessed by Cox-proportional hazard models. For the multivariate model building, we first included the presence of VF and those variables related to VF and to mortality according to the current literature (age, smoking and phosphate levels), and therefore these four variables were included in all the final models. Then we generated different models by entering the other predictors significantly associated with death in the univariate analysis (comorbidity, diabetes mellitus, overweight, diastolic blood pressure, eGFR, and serum 25 (OH) Vitamin D, 1,25 (OH) vitamin D, albumin and hemoglobin levels). Analyses were performed using SPSS statistical package version 15 (IBM, Armonk, NY, USA), and a *p*-value < 0.05 was considered significant. 

## 4. Results

Demographic and clinical characteristics are shown in Table 1. VFs were observed in 18% (*n* = 110) of the patients, with a total of 206 VFs, being the eleventh and twelfth thoracic vertebra (T11-12) and the first lumbar vertebra (L1) the most affected sites, corresponding to 62% of total VF (Table 2). Thus the median of VFs was 1 (1‒4). Patients with VF were significantly older and all of them presented arterial hypertension (Table 1). Prevalence of VF was analyzed according to age and categorized in 4 ranges: VF was present in 24.5 % of patients aged >76 years, while prevalence was 14.7% in those aged <60 years, 17.5% in 60‒68 years, and 15.3% in the group aged 69‒76 years. This difference did not reach statistical significance (ANOVA, *p* = 0.091). 

The distribution of VF according CKD stages was, 46 (41.8%) patients in stage 3, 50 (47%) patients in stage 4 and 14 (12.7%) patients in stage 5 NDD CKD. Regarding the severity of the VF (22), patients were discriminate in terms of number of fractures as mild in 73 (46.8%) patients; moderate in 70 (44.9%) patients and 28 (17.9%) with severe compromise.

Among biochemical variables, only low serum levels of phosphate and high levels of hsCRP were significant in patients with VF, who in turn tended to achieve in less proportion the normal ranges than patients without VF. Vascular calcification assessed by Adragao Score (hands only) tended to be more prevalent in patients with VF, although this difference did not reach significance. Treatment with bisphosphonates was more prevalent in the VF group.

In the univariate analysis, the group with VF was significantly older and presented lower serum phosphate levels, higher levels of hsCRP (*p* = 0.059), higher prevalence of ABI < 0.9, vascular calcification according Adragao (hands only) (*p* = 0.051) and lower compliance with K/DOQI recommendations. Binary logistic regression indicated that phosphate levels, ABI < 0.9, and treatment with bisphosphonates were independent factors associated with the presence of VF (Table 3). 

**Figure 2:**
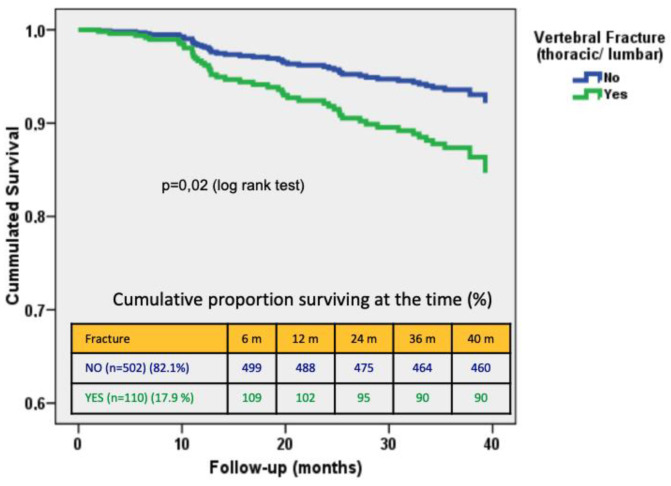
Kaplan Meier survival depending on the presence of vertebral fractures.

After a median follow-up of 35 months (IQR: 17‒37 months), sixty-two patients (10%) died, being the cardiovascular disease (*n* = 21; 34%) and infectious (*n* = 11; 18%) the leading causes of death Table 4). In the crude analysis, the group of fractured patients had poorer survival (log-rank test, *p* = 0.02; Figure 2). The VF severity was not associated with higher mortality (log-rank test, *p* = 0.392). After multivariate adjustment for age, eGFR, serum albumin, diabetes mellitus, comorbidity, Adragao Score > 3 and serum phosphate, the presence of VF (HR 1.983, 95% CI 1.009‒3.898, *p* = 0.047) was and independent predictor of all-cause mortality (Table 5). 

## 5. Discussion

This sub-analysis of the largest prospective study that explored the clinical outcomes and implications of vascular calcification in NDD CKD patients in stages 3‒5 reveals in 18% the presence of VF. Few studies in NDD CKD patients have focused on the prevalence of VF. We found that the prevalence of VF was similar to that reported in previous studies (19.4‒17%) [2,3]. However, these studies have not described clinical outcomes related to VFs in NDD CKD patients. In our findings, the factors associated to VF included age, peripheral vascular disease and, most strikingly, low serum phosphate levels. These results provide the first evidence that a decrease in blood flow of the lower extremities (measured as a decrease in the ankle/arm index) is associated with an increased risk of fractures in CKD patients; Laroche et al. [28], observed in 17 men with peripheral occlusive arterial disease, lower bone mineral density values and more VF than matched controls. Moreover, our findings are agreed with results from a community-based study where a decrease in the ankle/arm index was associated with an increase in the annual rate of bone loss [29]. Our observation is of interest, because recently there are several publications that suggesting a link between vascular disease and bone abnormalities [30,31], supporting the hypothesis that generalized atherosclerosis also affects bone circulation with reduced intraosseous circulation that may contribute to the development of osteoporosis [32,33], suggesting a link between peripheral artery disease and decreased bone mineral density. Correspondingly with our results, previous data suggested that serum biomarkers of bone metabolism are not clinically useful to assess the risk of fractures in patients with CKD [10,34]. Other investigation proved that pro-inflammatory cytokines (Il-1β, Il-6, IL-8 and TNF-α) accelerated bone loss and correlated well with some markers of bone turnover (PTH and osteocalcin) [35]. On the contrary, a novel finding of our study is the inverse correlation between serum phosphate levels with the presence of VF. The association of reduced serum phosphate levels with VF does not seem to be mediated by low PTH (not different in VF vs no-VF patients) or by differences in active serum vitamin D levels since serum calcitriol concentration were similar in VF vs no-VF. In this regard, low protein intake may contribute to a reduction in serum phosphate, poor nutrition, low grade inflammation and deficient muscle strength, all these factors are associated with VF. Certainly, this aggravated in older patients. It should be taken into consideration that in CKD patients most reports show that both high and low phosphate concentration are associated with increased mortality. In addition, our study confirms that elevation of serum phosphate is associated with vascular calcification and an aggravation of cardiovascular disease which contributes to mortality. This is not new; it provides certainty about our phosphate data. The observation of an association between low phosphate levels and VF is not incompatible with an association between high phosphate concentrations and mortality. Accepting these facts may help us to understand the importance of phosphate in kidney disease. Moreover, in kidney post transplants population, the association between some degrees of hypophosphatemia and fractures has been reported [36]. Remarkably, our results suggested that peripheral vascular calcification assessed by Adragao Score (hands) is related to VF. These findings are in agreement with previous studies in hemodialysis patients, which showed how the presence of vascular calcification in medium-sized and muscular arteries was significantly associated with VF [37]. Additionally, BMD seems to be associated with the progression of aortic calcifications in the general population [38,39,40]. These findings suggest that dysregulation in bone metabolism contributes to calcium deposits in arteries and may contribute to the pathogenesis of cardiovascular disease in CKD patients. However, details on the pathogenic mechanisms are beyond the purpose of this analysis and remain to be clarified. 

We found that VF are closely related to mortality in NDD-CKD stages 3‒5. Adjusted multivariate analysis revealed that the presence of VF nearly doubled mortality risk compared to CKD no-VF patients. The relationship between hip fractures with increased mortality and hospitalization in patients on dialysis was previously found [20], but the association of well- documented VF and mortality in NDD-CKD patients is a novel finding that warrants further research. In line with previous studies, hyperphosphatemia and vascular calcification were confirmed as independent factors for mortality [41,42]. In this regard, the fibroblast growth factor-23 (FGF23) may be involved in the pathogenesis of atherosclerosis and myocardial hypertrophy [43,44,45]. However, a few studies have been conducted to evaluate the effect of hypophosphatemia on cardiac function [46]. Thus, has been postulated that hypophosphatemia-induced cardiomyopathy and/or cardiac arrythmias and the mechanisms involved are a depletion of adenosine triphosphate (ATP) in myocardial cells and decreased 2,3-diphosphoglycerate (2,3-DPG) in erythrocytes [47,48]. Interestingly, this kind of cardiomyopathy can be potentially reversible.

The OSERCE 2 study, and subsequently this sub-analysis, had among its strengths the centralization of samples analysis and the radiologic reading, combined with a long period of follow-up. Moreover, the observational design does not allow the determination of outcomes related to prevention or treatment and other factors (physical activity, nutritional status, alcohol consumption). The approach of semiquantitative radiologic entails a limitation due to observer dependence. However, we minimized this factor by means of centralized and blind reading of X-rays undertaken by three independent expert radiologists. To exclude any selection bias, we compared baseline characteristics between patients excluded and included in this post-hoc analysis, not showing differences between groups.

In conclusion, our study revealed that VF are frequent and may be underdiagnosed in NDD-CKD patients. Factors associated with VF were age, low levels of phosphate and peripheral vascular disease. The presence of VF was an independent risk factor for mortality in these patients. Clinical trials are needed to confirm whether this relationship is causal and reversible with treatment for osteoporosis. 

## Figures and Tables

**Table 1:** Demographic and clinical characteristics of OSERCE 2 patients included.

Characteristics	Overall Sample (*n* = 612)	Vertebral Fractures (*n* = 110, 18%)	No Vertebral Fractures (*n* = 502, 82%)	*p*-Value
Age (y)	66.1 ± 12.7	68.0 ± 11.9	65.6 ± 12.9	0.043
Sex (male, %)	65.0%	60.0%	66.1%	0.133
BMI ≥ 30 (obese)	34.6%	36.4%	34.3%	0.722
BMI (kg/m^2^)	28.8 ± 5.1	28.7 ± 5.2	28.8 ± 5.1	0.974
Smoking	12.7%	9.4%	13.5%	0.425
Diabetes mellitus (%)	37.1%	33.6%	37.9%	0.232
Arterial hypertension (%)	94.4%	100.0%	93.6%	0.006
Mean bilateral ABI	1.0 ± 0.2	0.97 ± 0.2	1.0 ± 0.2	0.059
eGFR (MDRD mL/min/1.73 m^2^)	27.3 ± 11.6	27.5 ± 11.1	27.2 ± 11.7	0.823
Serum calcium (mg/dL)	9.8 ± 0.8	9.7 ± 0.8	9.6 ± 0.8	0.788
Serum phosphate (mg/dL)	3.5 ± 0.8	3.3 ± 0.8	3.5 ± 0.9	0.028
Serum iPTH(pg/mL) ^a^	61 (20‒123)	57 (17–107)	62 (80‒128)	0.437
K/DOQI Ca-target (%)	33.3%	30.2%	34.0%	0.449
K/DOQI P-target (%)	76.9%	78.3%	76.3%	0.708
K/DOQI PTH-target (%)	26.4%	24.5%	26.8%	0.628
All 3 K/DOQI crit. (%)	7.0%	2.8%	7.9%	0.065
Serum bicarbonate (mmol/L)	24.7 ± 3.5	24.4 ± 3.8	24.8 ± 3.4	0.440
C-reactive protein (mg/L) ^a^	2.0 (2.0–6.7)	3.8 (2.0‒7.7)	2.0 (2.0‒6.6)	0.012
Serum albumin (g/dL)	4.0 ± 0.5	3.9 ± 0.8	4.0 ± 0.5	0.243
Serum calcidiol (ng/mL)	20.5 ± 8.6	19.6 ± 7.6	20.7 ± 8.8	0.246
Serum calcitriol (pg/mL)	39.1 ± 10.4	39.7 ± 9.0	39.0 ± 10.6	0.498
Ca-based phosphate binder (%)	18.2%	14.7%	19.0%	0.180
Bisphosphonates (%)	3.0%	7.3%	2.0%	0.003
Adragao Score ≥ 3 (%)	30.9%	29.6%	31.2%	0.752
Adragao (hands only) >1 (%)	24.5%	31.4%	23.0%	0.075
Kauppila score > 6 (%)	31.4%	26.0%	32.6%	0.199

^a^ Skewed values are presented as median with inter-quartile range. Abbreviations: BMI, body mass index; ABI, ankle-brachial index; eGFR, estimated glomerular filtration rate; iPTH, intact parathyroid hormone; K/DOQI crit., complies with criterion/a. Bold numbers highlight significant *p*-values or those close to significance. If not indicated otherwise, results are presented as mean ± SD, or number (percent).

**Table 2:** Location of vertebral fractures.

Vertebrae	Vertebral Fractures (*n*,%)
T4	0 (0.0%)
T5	2 (1.0%)
T6	3 (1.5%)
T7	7 (3.5%)
T8	8 (4.0%)
T9	9 (4.5%)
T10	11 (5.5%)
T11	34 (17.0%)
T12	47 (23.0%)
L1	45 (22.0%)
L2	17 (8.0%)
L3	9 (4.0%)
L4	5 (2.0%)
L5	9 (4.0%)
Total vertebral fractures	T: 121 L: 85 (*n* = 206, 100%)

**Table 3:** Multivariate analysis for factors associated with the presence of vertebral fractures.

Factor	HR	95% CI	*p*-Value
Lower phosphate levels in blood	0.719	0.532−0.972	0.032
Ankle-brachial index <0.9	1.694	1.056−2.717	0.029
Treatment with bisphosphonates	5.636	1.876−16.930	0.002

The model included the variables found significantly related to vertebral fracture in the univariate analysis: age, C-reactive protein, use of anticoagulants or antihypertensive drugs, phosphoremia, ankle-brachial index < 0.9, Adragao Score (hands only) > 1, compliance of the three K/DOQI criteria, and treatment with bisphosphonates. HR, hazard ratio; CI, confidence interval.

**Table 4:** Death events and causes in patients with or without vertebral fracture.

Events	Fracture *n* = 110	No Fracture *n* = 502	*p*-Value
Total	21 (19%)	41 (8%)	0.05
Cardiovascular disease	7 (33%)	14 (34%)	0.38
Infectious disease	3	8	
Malignancy	1	6	
Sudden death	2	1	
Other	1	7	
Unknown	7	5	

There were 62 deaths (10%).

**Table 5:** Multivariate Cox-proportional hazard model for risk factors of mortality in CKD patients not on dialysis.

Factor	HR	95% CI	*p*-Value
Age	1.074	1.035‒1.114	<0.001
Adragao Score ≥3	2.487	1.345‒3.898	0.004
Phosphate levels	1.699	1.175‒2.488	0.005
Presence of vertebral fracture	1.983	1.009‒3.898	0.047

Multivariate analysis after adjusting by age, estimated glomerular filtration rate, serum albumin, presence of diabetes, Adragao Score >3 and phosphate levels.
